# A student-led pilot lecture in nutrition as a catalyst for curriculum change: A case study from one medical school

**DOI:** 10.1371/journal.pone.0336836

**Published:** 2026-05-29

**Authors:** Akash Patel, Ritika Modi, William McGonigle, Gauri Agarwal

**Affiliations:** 1 University of Miami Miller School of Medicine, Miami, Florida, United States of America; 2 University of Miami Miller School of Medicine, Division of Academic Medicine, Miami, Florida, United States of America; The Chinese University of Hong Kong, HONG KONG

## Abstract

**Background:**

Poor diet is the leading cause of global disability and premature death, contributing to 11 million deaths annually. Despite this, nutrition remains underemphasized in medical education, with over 70% of United States (U.S.) medical schools failing to meet the National Research Council’s 1985 recommendation of 25 hours of nutrition training. In 2022, the U.S. House of Representatives passed a bipartisan resolution calling for meaningful nutrition education for health professionals. Amid this gap, unreliable media sources frequently shape patient nutrition knowledge rather than professional sources such as registered dietitians or licensed physicians. Strengthening interprofessional collaboration with nutrition professionals could enhance dietary counseling and patient care.

**Methods:**

In response, one student implemented and evaluated the usefulness of a clinical nutrition lecture at the University of Miami Miller School of Medicine (UMMSM). Resulting student enthusiasm catalyzed the formation of a 50-member taskforce formed to expand and integrate nutrition longitudinally. A pre- and post-lecture survey around the session (Npre = 98, Npost = 77) assessed knowledge, perceptions of nutrition training, and comfort with dietary counseling.

**Results:**

Knowledge of evidence-based nutrition improved significantly (p < .001). Post-lecture, students reported greater confidence applying nutrition in clinical practice and increased interest in lifestyle medicine training (p < .001).

**Conclusions:**

After the session, there was increased self-reported confidence and understanding of nutrition’s role in health amongst students. Resulting student enthusiasm catalyzed the formation of a 50-member taskforce formed to expand nutrition education longitudinally with additional lectures to be integrated into the curriculum. This trajectory illustrates how even a small pilot can stimulate institutional dialogue and student-led momentum. We discuss key elements of an effective, multifaceted nutrition curriculum and propose a roadmap adaptable to other institutions.

## Introduction

Suboptimal diet is a leading contributor to global morbidity and mortality, associated with an estimated 11 million deaths and 255 million disability-adjusted life years (DALYs) worldwide in 2017 alone [1]. Poor dietary patterns are a major risk factor for noncommunicable diseases such as cardiovascular disease, type 2 diabetes, and certain cancers. In the U.S., dietary risks represent the largest individual risk factor for mortality, surpassing tobacco use, obesity, and other commonly cited health risks [2,3]. There exists a well-established association between unhealthy dietary patterns and reductions in both functional ability and life expectancy across populations [4].

Despite the clear impact of nutrition on health outcomes, medical education in the U.S. continues to underemphasize nutrition and dietary counseling. As early as 1985, the National Research Council recommended that medical schools provide at least 25 hours of nutrition education, yet over 70% of U.S. programs have failed to meet this benchmark. [5,6] At the same time, growing public interest in diet and preventive health has led to increased patient demand for accurate, evidence-based nutritional guidance [7].

Undergraduate medical education in the U.S. is accredited by the Liaison Committee on Medical Education (LCME), which does not require minimum hours of nutrition training or mandate applied instruction in counseling or chronic disease management [8]. Consequently, the amount and focus of nutrition teaching varies widely across schools. Much of the nutrition content traditionally encountered by medical students is biochemistry-focused (micronutrient metabolism and deficiency syndromes) but does not prioritize clinically oriented skills in counseling or diet-related chronic disease management [9]. Surveys consistently show insufficient preparation for evidence-based nutrition counseling and limited opportunities to practice applied skills [10,11].

Some medical schools have introduced nutrition electives, integrative medicine modules and teaching kitchen experiences [12]. However, these opportunities are often optional and inconsistently available, leaving many students without exposure to foundational nutrition training. Even when available, elective offerings vary widely in content, quality, and duration across institutions [9]. In a published 2025 report reflecting a nationwide survey, the Association of American Medical Colleges found that although 100% of schools included some nutrition in required curricula, fewer than half (45%) extended it across multiple courses, and only 17% achieved longitudinal integration across all years [13].

Expert groups and recent reviews have repeatedly noted this gap and called for greater standardization [10]. The recent JAMA consensus statement proposes 36 nutrition competencies and explicitly maps them to the Accreditation Council for Graduate Medical Education (ACGME) six core competency domains to facilitate integration across undergraduate and graduate training [14]. Recognizing this gap, the U.S. House of Representatives passed a bipartisan resolution in 2022 (H.Res.1118), calling for more comprehensive nutrition education for health professionals [14].

The American Heart Association (AHA) has echoed these concerns, noting that physicians without adequate nutrition training often lack the confidence to assess or counsel patients on dietary issues [11]. As a result, patients increasingly turn to unregulated media and social platforms for nutrition advice, sources that may propagate misinformation or even harmful guidance. The AHA advocates for the integration of longitudinal, systems-based nutrition education throughout medical curricula, with an emphasis on clinical relevance and interprofessional collaboration [15]. These recommendations also include structured learning alongside registered dietitians to promote both knowledge acquisition and appropriate referral practices.

In a wider context, it is important to acknowledge that improving physician training is only one part of the broader solution. Meaningful progress will also depend on changes to the food environment, including policies that expand access to affordable healthy foods, industry reforms, and stronger integration of registered dietitians into patient care. Expanding insurance coverage for medical nutrition therapy and establishing referral pathways are essential system-level needs. Within this wider lens, medical schools nonetheless have a clear and actionable opportunity to improve training.

In response to the recognized deficiency of formal nutrition education, one student worked with faculty to design and deliver a nutrition lecture integrated into and delivered during the cardiology block. Resulting positive student and faculty feedback spurred the formation of a student-led taskforce that is now collaborating with faculty to embed nutrition longitudinally at UMMSM with integrated nutrition lectures and other learning modalities in other pre-clinical blocks such as gastroenterology and endocrinology. This short report aims to evaluate our student-led pilot nutrition education initiative, showcase the catalytic effects of these efforts, and to propose a framework for longitudinal, competency-based integration of nutrition across medical training.

## Methods

We conducted a pre-post pilot intervention study to evaluate the immediate impact of a single clinical nutrition lecture, delivered as the first session of a planned multi-part lecture series at a single academic medical center during the 2023 academic year. Although the broader series was designed as a longitudinal curriculum, the present analysis is restricted to outcomes from the initial cardiovascular nutrition lecture, which functioned as a pilot and catalyst for subsequent curricular development.

All first-year medical students enrolled in the course (n = 192) were eligible to participate. Participation was voluntary, and no incentives were provided. Surveys were completed anonymously, and participation did not affect course evaluation or grades.

The lecture series was designed and delivered by one student with faculty oversight from clinicians and medical educators with expertise in nutrition, ensuring content accuracy and alignment with institutional curriculum. The 45-minute, in-person session focused on cardiovascular nutrition principles, including macronutrients, diet-related chronic disease management, and practical frameworks for patient dietary counseling. Teaching methods incorporated case-based learning, real-time polling, and small-group discussion to promote engagement and clinical application. Outcomes included a knowledge assessment and measures of self-reported confidence, attitudes, and preparedness, collected using structured pre- and post-lecture surveys. The knowledge assessment consisted of a 10-item instrument aligned with lecture objectives assessed both foundational nutrition knowledge and clinical application in cardiovascular disease prevention. Items included single-best-answer multiple-choice and true/false questions, with one point per correct response (range: 0–10). The instrument was developed for this study and has not been previously validated. The self-reported measures were assessed using 4-point Likert scale items (1 = not at all to 4 = a great deal) evaluating confidence in discussing nutrition, perceived importance of nutrition in clinical care, and preparedness to apply knowledge.

Pre-lecture surveys were administered electronically immediately prior to the session, and post-lecture surveys were distributed via QR code immediately afterward. Participants generated self-identifier codes to enable matching of responses. Due to incomplete or inconsistent identifiers, the final dataset included both matched and unmatched responses. Data were analyzed using two complementary approaches to address limitations in matching:

1) Unmatched analysis: All pre- and post-responses were compared as independent samples using Mann–Whitney U tests, given non-normal data distribution (Shapiro–Wilk p < 0.05). This approach allows population-level comparisons but does not assess individual-level change.
2) Matched analysis: For participants with linked pre- and post-responses, paired samples T-tests were used to evaluate within-subject changes in knowledge and self-reported measures. Despite non-normality, this test was selected based on sample size and absence of significant outliers.

Continuous and ordinal data are reported as medians with interquartile ranges (IQRs) for non-parametric analyses. For unmatched comparisons, Mann–Whitney U statistics (U), standardized test statistics (Z), p-values, and effect sizes (r) are reported. For matched analyses using paired-samples t-tests, results are reported as mean differences with standard deviations, 95% confidence intervals, t statistics, degrees of freedom (df), p-values, and corresponding effect sizes (Cohen’s d) to quantify the magnitude of within-subject change. Statistical significance was defined as p < 0.05. No a priori power calculation was performed due to the exploratory, pilot nature of the study. Analyses were conducted on available data without imputation.

All statistical analyses were performed using IBM SPSS Statistics, version 29.0. This study was approved by the University of Miami Institutional Review Board (Protocol #20231027) and determined to be exempt under 45 CFR 46.104 (Exempt Category 1: research conducted in established educational settings). Recruitment and data collection occurred on October 23, 2023, concurrent with lecture delivery. All participants provided informed consent electronically prior to participation. No identifying information was collected, and all data were analyzed in aggregate. All de-identified survey data are available in the Supporting Information (S1 Data), in accordance with journal data transparency requirements.

### Human ethics and consent to participate

This study was approved by the University of Miami Institutional Review Board (Protocol #20231027) and conducted in accordance with the principles of the Declaration of Helsinki. All participants provided written informed consent prior to participation through a digital form embedded in the pre-lecture survey. Participation was voluntary, and no identifying information was collected. The study followed the STROBE guidelines for reporting observational studies.

## Results

Of the 192 eligible first-year medical students, 98 completed the pre-lecture survey (response rate: 51%) and 77 completed the post-lecture survey (response rate: 40%). Matched pre–post responses were available for a subset of participants (n ≈ 58–63 depending on item), and both unmatched and matched analyses are reported. Knowledge assessment scores improved significantly following the lecture. In unmatched analyses (n_pre_ = 92, n_post_ = 77), median scores increased from 6 (IQR 5–7) pre-lecture to 9 (IQR 8–10) post-lecture (U = 862.0, Z = –8.622, p < .001, r = .66). In matched analyses (n = 61), mean knowledge scores increased from 5.72 (SD 1.22) to 8.42 (SD 1.23) (t(59) = –12.756, p < .001, d = –1.647, 95% CI [–2.033, –1.254]).

Self-reported knowledge of evidence-based nutrition increased significantly. Median scores rose from 2 (IQR 2–3) to 3 (IQR 3–4) (U = 1883.0, Z = –5.694, p < .001, r = .43). In matched analyses, mean scores increased from 2.46 (SD 0.88) to 3.31 (SD 0.62) (t(60) = –7.039, p < .001, d = –0.901, 95% CI [–1.197, –0.601]). Overall, both objective knowledge and self-reported knowledge of nutrition increased significantly following the lecture across analytic approaches.

*Confidence and Preparedness.* Confidence in applying nutrition principles to patient care improved significantly. In unmatched analyses (n_pre_ = 88, n_post_ = 77), median scores increased from 2 (IQR 2–3) to 3 (IQR 3–4) (U = 1702.5, Z = –5.847, p < .001, r = .46). Matched analyses (n = 58) showed mean scores increased from 2.38 (SD 0.77) to 3.14 (SD 0.66) (t(57) = –6.388, p < .001, d = –0.839, 95% CI [–1.136, –0.536]). These findings indicate a significant increase in students’ confidence in applying nutrition principles to clinical care.

*Attitudes Towards Nutrition and Medical Education*. Perceived importance of nutrition in disease management was high at baseline and did not significantly change in unmatched analyses (n_pre_ = 97, n_post_ = 77; median 4 [IQR 4–4] pre vs post; U = 3377.5, Z = –1.780, p = .075, r = .13). However, a small but statistically significant increase was observed in matched analyses (n = 63; mean 3.81 [SD 0.40] vs 3.92 [SD 0.27]; t(62) = –2.172, p = .017, d = –0.274, 95% CI [–0.524, –0.021]).

Interest in the inclusion of more lifestyle medicine in medical education increased significantly. In unmatched analyses (n_pre_ = 96, n_post_ = 77), medians remained at the ceiling (4 pre vs 4 post) but a statistically significant shift was noted (U = 2779.5, Z = –3.583, p < .001, r = .27). This was supported by matched analyses (n = 63; mean 3.57 [SD 0.64] vs 3.86 [SD 0.40]; t(62) = –3.582, p < .001, d = –0.451, 95% CI [–0.709, –0.190]).

Perceived confusion or contradiction in the nutrition space did not significantly change in unmatched analyses ((n_pre_ = 97, n_post_ = 77; median 4 [IQR 3–4] pre vs post; U = 3636.5, Z = –0.170, p = .865, r = .01), with no significant difference in matched analyses (n = 62; t(61) = 0.659, p = .256, d = 0.084). Overall, attitudes toward the importance of nutrition remained high, with increased interest in lifestyle medicine training but no change in perceived confusion in the nutrition space.

*Perceptions of Session Impact:* Post-lecture responses indicated that students perceived the session as impactful. The session influenced participants’ personal lifestyle choices (mean 3.26 [SD.71], median 3 [IQR 3–4]), was expected to influence future patient counseling (mean 3.52 [SD.60], median 4 [IQR 3–4]), and was rated as highly valuable overall (mean 3.80 [SD.46], median 4 [IQR 4–4]) [Tables 1–3].

## Discussion

### Impact of the pilot lecture: catalyzing institutional change

Findings demonstrate that even a single session can improve knowledge and confidence. More importantly, the pilot sparked momentum for broader change. Following overwhelmingly positive feedback, faculty invited the lecture to be repeated and expanded into other modules, and the success of the lecture series, combined with strong enthusiasm from peers, led to the formation of a 50-member student taskforce committed to advancing nutrition education. This taskforce, formed in the months following the initial lecture, now collaborates with faculty to design a longitudinal, systems-based curriculum at the UMMSM and is developing resources adaptable for medical students and health professionals nationally. The taskforce spans several institutions, has led to the development of a formal nutrition interest group at the UMMSM, and serves as a blueprint for student-driven change at other medical institutions. These findings demonstrate that the pilot achieved its aim of improving student knowledge and confidence while creating momentum for longitudinal curriculum reform.

These developments highlight how a small, student-led pilot can catalyze institutional reform when supported by faculty engagement and learner demand. At the same time, isolated lectures remain insufficient to close the gap in medical nutrition education and translate to improved public health. A lasting solution requires a curriculum that is mandatory, sustainable, and longitudinally integrated.

### Implementation options for nutrition education

The recent JAMA consensus statement identifies 36 nutrition competencies for medical trainees and maps them to the ACGME’s six core competency domains (patient care and procedural skills; medical knowledge; practice-based learning and improvement; interpersonal and communication skills; professionalism; and systems-based practice). Building on that framework, we propose a multifaceted, longitudinal model that operationalizes these competencies across the curriculum [14]. While several approaches occur concurrently rather than sequentially, layering them from pre-clinical through graduate medical education ensures competencies are introduced, reinforced, and applied in multiple clinical contexts. At UMMSM, this has taken shape through the following complementary strategies:

### Pre-learning resources

Foundational content can be delivered before class through concise, standardized resources. For example, self-paced online modules have proven effective in graduate medical education and can be completed independently and assessed internally [16]. One such external set of modules has been implemented at the UMMSM. In parallel, the UMMSM taskforce is developing short, interactive digital articles widely used in medical education, to introduce targeted nutrition concepts before in-class discussions [17]. *Primarily advances Medical Knowledge and Practice-Based Learning and Improvement* [14].

### Lectures and live teaching

Didactic sessions remain valuable for establishing frameworks and highlighting high-yield topics. Their effectiveness is greatest when paired with active elements such as polling or small group discussion. The UMMSM pilot lecture series, which began in cardiology and expanded into gastroenterology and endocrinology, illustrates how lectures can serve as effective entry points for broader curricular reform. *Primarily advances Medical Knowledge; when interactive, also supports Practice-Based Learning and Improvement* [14].

### Case-Based Learning (CBL)

CBL is already a core pedagogy in most U.S. medical schools, where students collaboratively analyze patient cases to build clinical reasoning. Embedding nutrition content into these cases normalizes dietary counseling as part of standard patient management. At UMMSM, nutrition questions are being incorporated into CBL scenarios for conditions such as diabetes and hyperlipidemia. Formal implementation is expected for the incoming class in 2026. *Primarily advances Patient Care and Procedural Skills and Practice-Based Learning and Improvement* [14].

**Table 1:** Unmatched analyses of pre- and post-lecture outcomes.

Outcome	Pre Median (IQR)	Post Median (IQR)	U	Z	P value	Effect size (r)
Knowledge and Confidence						
Knowledge Assessment Scores	6 (IQR 5–7)	9 (IQR 8–10)	862.0	−8.622	p < .001	.66
Self Assessment: How knowledgeable are you on the topic of evidence-based nutrition?	2 (IQR 2–3)	3 (IQR 3–4)	1883.0	−5.694	p < .001	.43
Self Assessment: How confident do you feel in your ability to apply nutrition principles to patient care?	2 (IQR 2–3)	3 (IQR 3–4)	1702.5	−5.847	p < .001	.46
Attitudes						
How valuable do you think nutrition is in disease management?	4 (IQR 4−4)	4 (IQR 4−4)	3377.5	−1.780	p = .075	.13
How much would you like to see the inclusion of more lifestyle medicine in medical education?	4 (IQR 4−4)	4 (IQR 4−4)	2779.5	−3.583	p < .001	.27
How much confusion and/or contradiction do you believe there to be in the nutrition space?	4 (IQR 3–4)	4 (IQR 3–4)	3636.5	−0.170	p = .865	r = .01

**Table 2:** Matched analyses of pre- and post-lecture outcomes.

Outcome	Pre Mean (SD)	Post Mean (SD)	t value	P value	Effect size (d, 95% CI)
Knowledge and Confidence					
Knowledge Assessment Scores	5.72 (SD 1.22)	8.42 (SD 1.23)	t(59) = –12.756	p < .001	–1.647, 95% CI [–2.033, –1.254]
Self Assessment: How knowledgeable are you on the topic of evidence-based nutrition?	2.46 (SD 0.88)	3.31 (SD 0.62)	t(60) = –7.039	p < .001	–0.901, 95% CI [–1.197, –0.601]
Self Assessment: How confident do you feel in your ability to apply nutrition principles to patient care?	2.38 (SD 0.77)	3.14 (SD 0.66)	t(57) = –6.388	p < .001	–0.839, 95% CI [–1.136, –0.536]
Attitudes					
How valuable do you think nutrition is in disease management?	3.81 (SD 0.40)	3.92 (SD 0.27)	t(62) = –2.172	p = .017	–0.274, 95% CI [–0.524, –0.021]
How much would you like to see the inclusion of more lifestyle medicine in medical education?	3.57 (SD 0.64)	3.86 (SD 0.40)	t(62) = –3.582	p < .001	–0.451, 95% CI [–0.709, –0.190]
How much confusion and/or contradiction do you believe there to be in the nutrition space?	3.45 (SD .69)	3.37 (SD .83)	t(61) = 0.659	p = .256	0.084, 95% CI [−.166, .333]

**Table 3:** Post-lecture perceptions of session impact.

Outcome	Median (IQR)	Mean (SD)
How much did the session influence your own lifestyle choices?	3 (3–4)	3.26 (.71)
How much will this session influence the way you advise patients in the future?	4 (3–4)	3.52 (.60)
How valuable did you find the session?	4 (4–4)	3.80 (.46)

### Standardized patients

Working with standardized patients (actors trained to simulate clinical encounters) provides a safe environment for students to practice motivational interviewing and personalize nutrition recommendations. These sessions also allow for structured feedback on counseling skills, though they require additional faculty time and institutional resources. At the UMMSM, a motivational interviewing session incorporating multiple modifiable risk factors, including diet, is expected to be implemented starting with the incoming class in 2026. *Primarily advances Interpersonal and Communication Skills and Professionalism; secondarily Patient Care* [14].

### Teaching kitchens

Teaching kitchens provide experiential, hands-on opportunities for students to learn nutrition through cooking and meal preparation. This model combines didactic instruction with practical skills, reinforcing concepts of healthy eating, food literacy, and patient counseling. At the UMMSM, a pilot culinary medicine elective has been developed to introduce students to evidence-based nutrition principles in an applied setting, while also highlighting strategies for patient engagement. Although resource-intensive, teaching kitchens can foster collaboration with dietitians and chefs and are increasingly recognized as a scalable modality for nutrition education. This experiential component, which integrates interprofessional collaboration, is expected to be offered as a formal elective for the incoming class in 2026. *Primarily advances Interpersonal and Communication Skills and Professionalism; secondarily Patient Care* [12,14].

### Graduate medical education

Nutrition training should extend beyond medical school. Specialty-specific content in residency and fellowship ensures that nutrition is reinforced when it is most clinically relevant and allows tailoring to specialty populations (e.g., cardiovascular disease, cancer survivorship, or chronic kidney disease). *Primarily advances Systems-Based Practice* [11,14].

**Fig 1:**
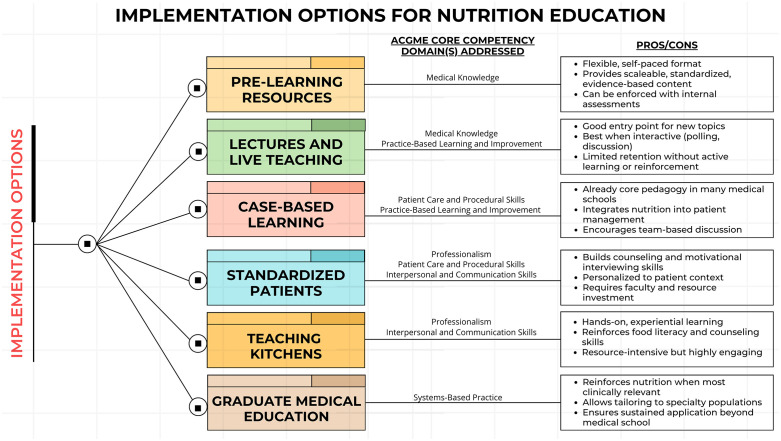
Implementation options for nutrition education and the ACGME competency domains they address. Pathways include pre-learning resources, lectures and live teaching, case-based learning, standardized patients, teaching kitchens, and graduate medical education. Each approach lists the primary ACGME domain(s) addressed (Medical Knowledge; Patient Care and Procedural Skills; Practice-Based Learning and Improvement; Interpersonal and Communication Skills; Professionalism; Systems-Based Practice) alongside illustrative pros/cons. Approaches may occur concurrently rather than sequentially; layering them across training reinforces and applies nutrition competencies in diverse contexts.

These components are most impactful when layered together, and each advances different domains within the ACGME core competency framework. In Fig 1, we annotate every implementation option with the primary ACGME domain(s) it addresses to show how the model operationalizes the JAMA consensus nutrition competencies across the curriculum [14].

### Towards standardization and broader adoption

Wider adoption across institutions will ultimately depend on standardization. The recently proposed core competencies in nutrition education offer a roadmap for curriculum design, evaluation, and faculty development. Since these competencies are categorized within the ACGME’s six core domains, linking implementation strategies and assessments to those domains can streamline integration with existing milestones, entrustable professional activities (EPAs), and program evaluation [14]. National medical education organizations and accrediting bodies can help promote consistency and support quality control across institutions. Aligning implementation strategies with recent policy trends may facilitate broader uptake and sustained momentum. The AAMC report highlighted that only 17% of medical schools achieve longitudinal integration, underscoring the urgency of operationalizing nutrition competencies through structured, multi-modal strategies [13].

### Role of physicians and dietitians

While registered dietitians are the recognized experts in nutrition care and should be prioritized as central members of the healthcare team, there remain systemic barriers to fully leveraging their expertise. Coverage for medical nutrition therapy is limited, referral pathways are inconsistent, and dietitians are not available in all care settings [11]. In this context, equipping physicians with foundational nutrition training is not intended to supplant the role of dietitians, but rather to complement and amplify it. Physicians remain the first point of contact for most patients, and as central coordinators of care, they have a unique opportunity to influence patient perceptions and behaviors. Even brief, evidence-based nutrition guidance from physicians can help normalize dietary counseling as part of standard medical care and can facilitate timely referrals to dietitians. Moreover, physician training in nutrition may strengthen advocacy for expanded access to dietitian services. Medical trainees often become key decision makers in health systems and health policy; their understanding of nutrition’s role in patient health is essential to addressing systemic change. Our survey finding that many students reported obtaining nutrition information from social media further underscores this need, as patients are likely navigating similar information environments and may rely on physicians to contextualize or correct conflicting guidance. Importantly, any nutrition education added to the medical curriculum must be carefully considered within the already crowded landscape of medical training, continued throughout residency and fellowship where it has the greatest clinical relevance, and focused on the practical skills needed to work effectively alongside dietitians and other professionals.

### Limitations

This study evaluated a single lecture at a single institution with voluntary participation, modest response rates, and anonymous surveys that prevented reliable matching of pre- and post-responses. Outcomes were short-term and largely self-reported, and the assessment tool was not formally validated. The unmatched pre-post design further limits causal inference. Results may be influenced by response bias and may not generalize to other settings, and no long-term follow-up was conducted to assess retention or behavioral change. Additionally, the proposed implementation “roadmap” is conceptual and was not formally evaluated. Future work should include multi-site pilots, longitudinal retention and patient-level outcomes.

## Conclusion

Strengthening physician training in nutrition is a necessary step toward addressing the growing burden of diet-related disease. While our findings are limited to the first lecture in a three-part series, the impact extended beyond measurable knowledge gains: faculty implemented additional nutrition objectives and student enthusiasm led to the creation of a 50-member taskforce dedicated to further curriculum reform. This illustrates how even a small pilot can evolve into a multifaceted student-driven model for institutional change. With institutional support and alignment with national competencies, medical education can begin to close the gap between what patients need and what future physicians are prepared to deliver. Such training should be viewed as complementary to, not a replacement for, the expertise of registered dietitians. Equipping physicians with baseline skills can also help strengthen collaboration and advocacy for expanded access to dietitian services.

## Supporting information

S1 Data De-identified pre- and post-lecture survey dataset. (XLSX)

S1 File Knowledge assessment items administered pre- and post-lecture. (DOCX)
