# Supplementary material for: A student-led pilot lecture in nutrition as a catalyst for curriculum change: A case study from one medical school
Source: PLoS One. 2026 May 29;21(5):e0336836. doi: 10.1371/journal.pone.0336836 (PMC13221018; doi:10.1371/journal.pone.0336836)
Supplement: S1 File — (DOCX) [file pone.0336836.s002.docx]

Supplementary Material: Knowledge Assessment Items Administered Pre- and Post-Lecture

1. Which of the following dietary patterns has not been implicated in improved long-term health outcomes, specifically with respect to cardiovascular disease?
   1. Mediterranean Diet
   2. Ketogenic Diet
   3. Whole-Food Plant-Based Diet
   4. DASH Diet
2. Nutrition science has identified a single optimal diet for human health (T/F).
3. “Cutting carbs” is generally a strong and often necessary recommendation for patients (T/F).
4. Due to their high fat content, high consumption of nuts is generally associated with worse cardiovascular disease outcome (T/F).
5. Swapping out saturated fats for which of the following into the diet has been associated with the greatest reduction in disease-specific and all-cause mortality?
   1. Polyunsaturated Fatty Acids
   2. Monounsaturated Fatty Acids
   3. Trans Fatty Acids
   4. Refined Grains
6. What percentage of the U.S population is estimated to not reach sufficient fiber goals?
   1. 30-35%
   2. 60-65%
   3. 80-85%
   4. 90-95%
7. Per 1000 kilocalories, which of the following is recommended target for fiber intake?
   1. 5 grams
   2. 14 grams
   3. 19 grams
   4. 30 grams
8. Which of the following is relevant to nutrition counseling?
   1. Socioeconomic status
   2. Current diet
   3. Patient goals
   4. Accessibility
   5. Culture
   6. All of the above
   7. Only B & C
9. Early signs of atherosclerotic processes (such as fatty streaks and LDL oxidation) have been shown as early as:
   1. Fetal development
   2. First decade of life
   3. Second decade of life
   4. Third decade life
   5. Fourth decade of life
10. According to dietary guidelines, which of the following is an appropriate intake of saturated fat for the average individual?
    1. No amount of saturated fat is okay
    2. Less than 5% of total energy intake
    3. Less than 10% of total energy intake
    4. Less than 15% of total energy intake
    5. There is no limit to saturated fat intake
